# Prevalence of alcohol and other psychoactive substance abuse and association with depression among medical students in Niger Delta University, Bayelsa State

**DOI:** 10.11604/pamj.2024.47.90.35997

**Published:** 2024-02-27

**Authors:** Tamaraemumoemi Emmanuella Okoro, Uzoechi Eze Chikezie

**Affiliations:** 1 Department of Internal Medicine, Niger Delta University Teaching Hospital, Okolobiri, Bayelsa State, Nigeria

**Keywords:** Depression, medical students, alcohol use, substance abuse

## Abstract

**Introduction:**

alcohol and other psychoactive substances have adverse health effects, particularly on young people. This study determined the prevalence of alcohol and other psychoactive substance abuse and its association with depression among Niger Delta University, Bayelsa State, Nigeria, medical students.

**Methods:**

a cross-sectional study involving 243 medical students who completed a patient-rated version of the Mini International Neuropsychiatric Interview (MINI-PR). For analyzing the data, descriptive and inferential statistics were employed.

**Results:**

most respondents were 18 to 24 years old (67.1%), and 52.7% were male; the prevalence of major depressive episodes (current) and lifetime alcohol and other psychoactive use was 30.5%, 25.5%, and 21%, respectively. Also, the prevalence of current alcohol abuse and dependence was 5.8% and 4.9%, respectively. Alcohol use (χ^2^: 12.57, p = 0.001) and abuse (χ^2^: 22.33, p = 0.001) were significantly associated with depression. Psychoactive substance use was significantly associated with depression (χ^2^: 12.91, p = 0.001). The odds of having depression increased with the use of alcohol (OR: 3.54; 95% CI: 1.71-7.33) and psychoactive substances (OR: 4.52; 95% CI: 1.88-10.88).

**Conclusion:**

alcohol and psychoactive substance use were significantly associated with depression. Organizing interventions to reduce such unhealthy social practices among medical students is necessary.

## Introduction

Psychoactive substances are chemical substances that can change an individual´s consciousness, mood, and thinking processes when taken [1]. Psychoactive substances include alcohol, caffeine, nicotine, marijuana, and illegal drugs, such as heroin, lysergic acid diethylamide (LSD), cocaine, and amphetamines [2]. Alcohol is a socially accepted psychoactive substance and may serve as a 'gateway' to using other psychoactive substances. Young people begin experimenting with alcohol and cigarettes mainly from influences that include peer pressure, advertisements, dysfunctional home settings, and the inability to cope with stress [2].

Medical education is highly stressful, and this stressful academic environment can negatively affect medical students' physical and psychological well-being [3]. One primary source of stress for medical students is the amount and complexity of the materials they must assimilate in the course of study [4]. Contributory factors include fear of failure, family issues, academic performance uncertainties, and meeting their teachers´/lecturers´ expectations [5]. Medical students may resort to psychoactive substances to enhance physical and cognitive performance to cope with the enormous challenges they face in their studies [6].

Medical students are future medical practitioners and may potentially treat patients with substance use disorders. Alcohol and other psychoactive substances use are associated with depression, suicidal ideation, aggressiveness, self-harm, and alcohol dependency [6]. Studies show an increased risk of depression in medical students compared to other university undergraduates and the general population [7,8]. Studies assessing alcohol and psychoactive substance use and its association with depression in medical students in our environment are scarce. Makanjuola *et al*. [6] assessed psychoactive substance use among medical students in a Nigerian university and found that the most currently used substances were mild stimulants such as coffee (33.3%). Others were alcohol (13.6%), sedatives (7.3%), and tobacco (3.2%). However, they did not assess for depression in their respondents. Several other studies have evaluated the prevalence of depression in medical students [9,10] or alcohol and other psychoactive substance use [6,11,12]; however, studies exploring the association between depression and alcohol and other psychoactive substance use in medical students are scarce. Suraj *et al*. [13] discovered no link between depression and the use of psychoactive drugs in their study of the prevalence and variables associated with depression among medical students at a Northern Nigerian institution (p = 0.311). Their finding may be due to their study respondents' low prevalence of psychoactive substance use. Out of the 279 study participants in the study by Suraj *et al*. [13], only seven admitted to psychoactive substance use.

Our study was conceptualized to assess the prevalence of depression, alcohol, and psychoactive substance use among medical students and investigate the relationship between alcohol/psychoactive use and depression among these students in Niger Delta University, Bayelsa State, Nigeria. The research questions were: 1) what is the prevalence of depression and alcohol/psychoactive use among medical students in NDU, Amasoma? 2) Is there a relationship between depression and alcohol/psychoactive use among medical students in NDU, Amasoma? 3) What is the relationship between sociodemographic characteristics/academic performance and depression among medical students in NDU, Amasoma? We hypothesize that “there is no relationship between depression and the use of alcohol/psychoactive substance among medical students in NDU, Amasoma”.

## Methods

**Study area:** the study was conducted among preclinical and clinical students of the College of Health Sciences, Niger Delta University (NDU), Amassoma. The Bayelsa State government established the Niger Delta University (NDU) in 2000. The University is situated on Wilberforce Island, Amasoma, about 30 kilometers from the State capital, Yenagoa. The College of Health Sciences campus was also created in 2000 and is the oldest campus in the university.

**Study design:** the study was a descriptive cross-sectional study. Data collection occurred for 2 months (January to February 2020).

**Study population:** the study involved all medical students at all levels of study (pre-clinical and clinical) who gave informed consent to participate. Only students who gave voluntary consent were recruited.

**Sample size:** utilizing the formula for the estimation of single proportions [14], as stated below, the sample size was determined with the prevalence of current alcohol use among Nigerian university students (33%), as reported by Adayonfo *et al*. [12], which gave a value of 390 participants. The minimum sample size was then adjusted using a correction formula for the desired sample size calculation for a finite population of less than 10,000 and further adjusted for non-response using a non-response rate of 20% to obtain a sample size of 203 persons [15,16]. However, we eventually sampled 243 medical students at Niger Delta University, Amasoma, Bayelsa State.

**Sampling technique:** a probability sampling technique, simple random sampling (balloting), was used to recruit participants from each level of the study (years 1 to 6). The class attendance register was used as the sampling frame, and the student number on the register was taken as each participant´s unique identifier for the study.

**Study instrument:** this study's instrument was a 2-section questionnaire developed by the researchers with 61 items comprising validated tools used in different settings to assess depression and associated factors. Section one of the study tool collected data on socio-demographic characteristics, level of study, living arrangement, accommodation conditions, socioeconomic class, and participants' academic performance. Section two contained the relevant portions of the patient-rated Mini International Neuropsychiatric Interview (MINI-PR) version 5.0.0 [17]. Part A1 - A6 of the MINI-PR explored depression; part J1 - J3 investigated alcohol disorder/abuse, while part K1 - K3 examined non-alcohol substance disorder/abuse. The MINI-PR has been widely used and validated in assessing depression and psychoactive substance/alcohol use and abuse in African studies [18,19].

**Study procedure:** two research assistants were medical interns working in the Internal Medicine Department of the Niger Delta University Teaching Hospital were recruited as research assistants and trained by the principal investigators to collect the data alongside the two principal investigators. The different items on the questionnaire were explained to the research assistants to ensure they understood the response each item was designed to elicit. Also enumerated during the training were the objectives and procedure for the study and how to obtain consent for the study. Before the data collection, the class representatives at each grade level were also informed about the study´s objectives, purpose, and benefits. In turn, they helped organize class members who had the questionnaires administered to them during their breaks. The questionnaires were self-administered by the recruited participants of each grade level. Each returned questionnaire was cross-checked to make sure it was filled in correctly. Each questionnaire took approximately 15 minutes to complete. The research assistants and the principal researchers were available to assist participants, when necessary, in filling out the questionnaires. Confidentiality and anonymity were upheld. The questionnaires were pretested before the commencement of the study among 3^rd^-year medical students at the University of Benin, Nigeria, to ensure the validity and reliability of the questionnaire.

**Data analysis:** data were coded, entered, cleaned, and analyzed on Statistical Package for Social Sciences (SPSS version 23) software [20]. Categorical variables like sex, marital status, socioeconomic status, presence of depressive episodes, alcohol, and psychoactive use were summarized as frequencies and percentages; age, a continuous variable in the study, was summarized as mean and standard deviation. The decision on the presence of depressive episodes, alcohol, non-alcohol psychoactive substance disorder, and abuse was taken based on the guide in the relevant sections of the Mini International Neuropsychiatric Interview (MINI version 5.0.0) used in the study. After identifying participants with depressive episodes, alcohol, and psychoactive substance abuse/disorder, the proportions of these conditions were calculated to obtain the prevalence among medical students. The relationship between alcohol, substance use/abuse, living arrangement, sociodemographic/academic characteristics, and depression was explored using the Chi-square tests. Binary logistic regression analysis was carried out to investigate further the strength of the relationship between the dependent variable (depressive episodes) and the independent variables (Gender, age, living arrangement, year of study, self-rated academic performance, academic failure experience, alcohol/psychoactive use) in the study the significance level was set at ≤ 0.05.

**Ethical issues:** the study was conducted per the Helsinki Declaration [21], and ethical clearance ((application form no NDUTH REC/0045/2017) was obtained from the Research and Ethics Committee of the Niger Delta University Teaching Hospital. All participants in the study gave their voluntarily signed informed consent. All data was handled with strict confidentiality.

## Results

**Sociodemographic characteristics of participants:** over half (52.7%) of the 243 participants in the study were male medical students, and 115 participants (47.3%) were females. The mean age of participants was 24.2 ± 5.1 years. Table 1 further revealed that most participants were aged 18 to 24 years (67.1%), single (94.2%), and Christian (97.5%).

**Table 1:** sociodemographic characteristics of study participants

Variable	Frequency (N = 243)	Percent (%)
**Gender**		
Female	115	47.3
Male	128	52.7
**Age group**		
18 - 24 years	163	67.1
25 - 29 years	51	21.0
30 - 34 years	15	6.2
≥ 35 years	14	5.8
**Age in years - mean ± SD**	24.2 ± 5.1	
**Marital status**		
Married	14	5.8
Single	229	94.2
**Religion**		
Christian	237	97.5
Others a	6	2.5
**Socioeconomic status**		
High	10	4.1
Middle	193	79.4
Low	40	16.5

N = Total sample size; ^a^Others - including Islam, African traditional religions (ATR)

**Living arrangement and academic features among study participants:** one hundred and two participants (42.0%) live with other students in accommodation provided by the school management, and about a quarter (24.7%) live alone on campus. Ten students (4.1%) live off campus with their parents. Most students (24.3%) were in their first year of study (Table 2). One hundred sixteen students (47.7%) and 105 (43.2%) rated their academic performance as 'good' and 'average', respectively. About a third of participants (32.9%) had failed one academic examination in medical school (Table 2).

**Table 2:** living arrangements and academic features among study participants

Variable	Frequency (N = 243)	Percent (%)
**Living arrangement**		
Live in with other students on campus	102	42.0
Live alone on campus	60	24.7
Live alone off campus	50	20.6
Live with other student off campus	21	8.6
Live with parents of campus	10	4.1
**Year of study**		
1	59	24.3
2	26	10.7
3	35	14.4
4	47	19.3
5	25	10.3
6	51	21.0
**Academic performance**		
Excellent	16	6.6
Good	116	47.7
Average	105	43.2
Below average	3	1.2
Poor	3	1.2
**The student has experienced academic failure in the medical school**		
Yes	80	32.9
No	163	67.1

N = Total sample size

**Prevalence of depression, alcohol, and psychoactive substance use:** as shown in Table 3, the prevalence of depression, alcohol use, and use of psychoactive substances, as defined by the MINI questionnaire, was 30.5%, 14.8%, and 9.9%, respectively. Alcohol abuse and psychoactive substance disorder were seen in 5.6% and 5.3% of participants, respectively (Table 3).

**Table 3:** depression, alcohol, and psychoactive substance use/disorder as classified by MINI scale among study participants

Characteristics	Frequency (n = 243)	Percent (%)
**Depression**		
Major depressive episode, current	74	30.5
Major depressive episode, recurrent	70	28.8
Depression with melancholic features	65	26.7
**Alcohol abuse and dependence**		
Alcohol use	36	14.8
Alcohol abuse, current	14	5.8
Alcohol dependence, current	12	4.9
**Psychoactive substance use disorder**		
Psychoactive substance use	24	9.9
Psychoactive substance disorder	13	5.3
Psychoactive substance abuse	9	3.7
Psychoactive substance dependence	5	2.1

**Association between alcohol, psychoactive substance use, and depression:** twenty students of the 74 students who had depressive episodes (27.0%) took alcohol, while only 16 of 169 students (9.5%) without a depressive episode in the two weeks preceding the survey took alcohol; this shows a significant relationship between the occurrence of depressive events and the use of alcohol (χ^2^= 12.57; p - 0.001) among participants in this study. Furthermore, the odds of depression were three times higher (odd ratio - 3.54; 95%CI: 1.71 - 7.33) among students who used alcohol than those who did not (Table 4). Fifteen of the 74 students who had depressive episodes (20.3%) used psychoactive substances, while 9 of 169 students (5.3%) without depressive episodes used psychoactive substances, also showing a significant relationship between depressive episodes and psychoactive substance use (χ^2^= 12.91; p - 0.001). It is four times more likely that students using psychoactive substances would have experienced depressive episodes (odd ratio - 4.52; 95%CI: 1.88 - 10.88) in the two weeks preceding this survey (Table 4).

**Table 4:** relationship between depression and alcohol, substance abuse/disorder among study participants

Characteristics		Depression		χ^2^; (p-value)	OR (95% CI)
	Total N = 243 (%)	Yes N = 74 (%)	No N = 169 (%)		
**Alcohol use**					
Yes	36 (14.8)	20 (27.0)	16 (9.5)	12.57 (0.001*)	3.54 (1.71 - 7.33)
No	207 (85.2)	54 (73.0)	153 (90.5)		1
**Alcohol dependence**					
Dependence	12 (4.9)	11 (14.9)	1 (0.6)	22.33 (0.001*)	29.33 (3.71 - 231.8)
No dependence	231 (95.1)	63 (85.1)	168 (99.4)		
**Psychoactive substance use**					
Yes	24 (9.9)	15 (20.3)	9 (5.3)	12.91 (0.001*)	4.52 (1.88 - 10.88)
No	219 (90.1)	59 (79.7)	160 (94.7)		1
**Psychoactive substance disorder**					
Disorder	13 (5.3)	8 (10.8)	5 (3.0)	6.28 (0.012*)	3.98 (1.26 - 12.60)
No disorder	230 (94.7)	66 (89.2)	164 (97.0)		1
**Psychoactive substance dependence**					
Dependence	5 (2.1)	4 (5.4)	1 (0.6)	5.92 (0.031*)	9.60 (1.05 - 87.42)
No dependence	238 (97.9)	70 (94.6)	168 (99.4)		1

* Statistically significant; OR: odd ratio; CI: confidence interval

**Relationship between sociodemographic characteristics, living arrangement, academic performance, and depression among study participants:** Table 5 showed that poor and below-average performing students were 21 times more likely to have depression when compared to excellent students (odd ratio - 21.0; 95%CI: 1.40 - 314.0). Students who experienced academic failure showed an increased chance of developing depression (odd ratio - 1.92; 95%CI: 1.02 - 3.39). The living arrangement of students was also significantly related to experiencing depression among the medical students (χ^2^= 11.43; p -0.02). Although not statistically significant, the odds of depression were higher among female medical students (odd ratio - 1.73; 95%CI: 0.99 - 3.00) than their male counterparts (Table 5).

**Table 5:** relationship between sociodemographic characteristics, living arrangement, academic performance, and depression among study participants

Characteristics		Depression		χ^2^; (p-value)	OR (95% CI)
	Total N = 243 (%)	Yes N = 74 (%)	No N = 169 (%)		
**Sex**					
Female	115 (47.3)	42 (56.8)	73 (43.2)	3.80 (0.051)	1.73 (0.99 - 3.00)
Male	128 (52.7)	32 (43.2)	96 (56.8)		1
**Age group**					
18 - 24 years	163 (67.0)	57 (77.0)	106 (62.7)	5.97 (0.113)	3.23 (0.70 - 14.92)
25 - 29 years	51 (21.0)	13 (17.6)	38 (22.5)		2.05 (0.41 - 10.41)
30 - 34 years	15 (6.2)	2 (2.7)	13 (7.7)		0.92 (0.11 - 7.62)
≥ 35 years	14 (5.8)	2 (2.7)	12 (7.1)		
**Living arrangement**					
Live in hostel	102 (42.0)	38 (51.4)	64 (37.9)		2.71 (1.19 - 6.18)
Live alone	60 (24.7)	13 (17.6)	47 (27.8)	11.43 (0.022*)	1.26 (0.49 - 3.25)
Live with student	21 (8.6)	10 (13.5)	11 (6.5)		4.14 (1.35 - 12.69)
Live with parents	10 (4.1)	4 (5.4)	6 (3.6)		3.04 (0.71 - 31.03)
Live outside	50 (20.6)	9 (12.2)	41 (24.3)		1
**Academic performance**					
Excellent	16 (6.6)	2 (2.7)	14 (8.3)	18.02 (0.001*)	1
Good	116 (47.7)	30 (40.5)	86 (50.9)		2.44 (0.52 - 11.38)
Average	105 (43.2)	36 (48.6)	69 (40.8)		3.45 (0.74 - 16.05)
Below average	3 (1.2)	3 (4.1)	0 (0.0)		21.00 (1.40 - 314.0)
Poor	3 (1.2)	3 (4.1)	0 (0.0)		21.00 (1.40 - 314.0)
**Student has experienced academic failure in the medical school**					
Yes	80 (32.9)	32 (43.2)	48 (28.4)	5.13 (0.023*)	1.92 (1.09 - 3.39)
No	163 (67.1)	42 (56.8)	121 (71.6)		1

* Statistically significant; OR: odd ratio; CI: confidence interval

## Discussion

We examined the prevalence of alcohol and other psychoactive substance abuse and its association with depression among medical students attending Niger Delta University, Bayelsa State, Nigeria. Our results show a 14.8% prevalence of alcohol use and a 9.9% prevalence of other psychoactive substance use. The prevalence of alcohol use in our study was within the range of the prevalence of alcohol previously reported among medical students in Nigeria [6,22]. Several studies show that psychoactive substance use among medical students ranges from 4% to 40.4% [6,7,22,23]. Differences in the prevalence of alcohol use and psychoactive substance use in various studies, including ours, may reflect study participants' socio-cultural characteristics and methodology [24].

The prevalence of depression among our study participants, defined by the MINI questionnaire, was 30.5%. Several studies show that medical students suffer an increased risk of depression compared to other students and the general population [25,26]. They consistently show higher depression, anxiety, and stress scores than the general population [25,26]. The reason for the high vulnerability of medical students to depression has been linked to personality traits such as an increased conscientiousness, which on the one hand, enhances academic performance, but on the other hand, may also render them highly vulnerable to self-criticism and self-doubt in an environment of high academic demand [25]. Medical training is a major psychological stressor, and there are numerous replications of the observation that medical students have higher depression scores than comparable age-matched groups [25-27].

Our study found that alcohol use/dependence and psychoactive substance use were significantly associated with depression among the respondents. Our findings are similar to previous findings that show medical students have high levels of depression, anxiety, and stress compared with the general population [25-27], and past-month use of alcohol, including lifetime use of illicit substances and or alcohol, has been significantly associated with depression in them [28]. Certain factors may predispose to depression in medical students. Our study quantified the risk of developing depression concerning academic performance. Examination failures were associated with an increased risk of depression (OR - 1.92; p - 0.024). Poor and below-average performing students were 21 times more likely to have depression when compared to excellent students. Our finding agrees with other studies that show medical students may face higher levels of education-related stressors and have higher levels of psychological (depression morbidity than age and sex-matched peers in different academic disciplines [29,30]. These contributory educational factors include the vastness of the academic/medical curriculum, fear of poor performance or actual performance in examinations, lack of recreational activities, frequency of continuous assessments/examinations, competition with peers, high family/parental expectations, and psychosocial factors [30].

**Limitation:** the responses were self-reported. This could have led to recall bias.

## Conclusion

Alcohol and psychoactive substance use were significantly associated with depression. There is a need to organize interventions to reduce such unhealthy social practices among medical students.

### What is known about this topic

- *Medical students show an increased risk of depression compared to other university undergraduates and the general population;*
- *Medical students may resort to psychoactive substances to enhance physical and cognitive performance to cope with the enormous challenges they face in their studies*.

### What this study adds

- *Alcohol use/dependence and psychoactive substance use are significantly associated with depression among medical students;*
- *The prevalence of depression is high in medical students, and those with poor academic performance were 21 times more likely to have depression when compared to students with excellent performance*.
